# Energy Drinks and Sleep among Adolescents

**DOI:** 10.3390/nu14183813

**Published:** 2022-09-15

**Authors:** Milena Tomanic, Katarina Paunovic, Maja Lackovic, Katarina Djurdjevic, Milica Nestorovic, Ana Jakovljevic, Milos Markovic

**Affiliations:** 1 Institute of Hygiene and Medical Ecology, Faculty of Medicine, University of Belgrade, 11000 Belgrade, Serbia; 2 Faculty of Medicine, University of Belgrade, 11000 Belgrade, Serbia; 3 Clinic for Psychiatry, University Clinical Centre of Serbia, 11000 Belgrade, Serbia; 4 Faculty of Medicine, Institute for Pharmacology, Clinical Pharmacology and Toxicology, University of Belgrade, 11000 Belgrade, Serbia; 5 Clinic for Mental Disorders “Dr Laza Lazarevic”, 11000 Belgrade, Serbia

**Keywords:** energy drinks, sleep, adolescents, caffeine, mental health, diet, physical activity

## Abstract

Many adolescents worldwide have the problem of meeting recommended nightly sleep hours. The causes of sleep disturbance are multifactorial, but interest in food’s effect on sleep has dramatically increased lately. In this study, we investigated the association between regular energy drink (ED) intake (weekly or more frequent) and sufficient sleep (SS) (≥8 h) in adolescents. Additional objectives were to examine the relationship between health-related behaviors and SS, stratified by gender. A population-based cross-sectional study was conducted during the 2019/2020 school year from 12 schools in Belgrade. There were 1287 students aged 15 to 19 who participated (37.4% male). We used a modified version of the food frequency questionnaire adapted for Serbian adolescents. Logistic regression revealed that regular ED consumption was an independent risk factor negatively related to SS in both sexes. Additionally, daily vegetable and water intake (≥2 L) showed a positive correlation with SS in boys, while in girls, the odds of realizing SS decreased with statements of sedative use. In conclusion, we show that ED intake is negatively associated with SS in both sexes; daily vegetable and water intake (≥2 L) may raise the odds of SS in boys, while sedative use may decrease the chances of SS in girls.

## 1. Introduction

Adolescence is a period of great importance in human development, characterized by the accelerated maturation of physical, psychological, and cognitive function. A few requirements must be satisfied to support these processes, including optimal internal environment (absence of disease) and optimal external conditions (lack of harmful influences from surroundings) [1]. It is well known that most critical neurobiological and hormonal processes occur during sleep time [2]. The role of sleep in maintaining physical and mental health, and general well-being in adolescence, is unambiguous [3].

A panel of thirteen sleep experts supported by the American Academy of Sleep Medicine and the Centers for Disease Control (CDC) in 2016 came to a common consensus regarding recommended night sleep length in children and adolescents [4]. According to those recommendations, teenagers aged 13 to 18 should sleep 8 to 10 h during a 24 h period to maintain optimal health.

However, night sleep shortening in adolescence is evident in many countries [5,6]. Teenage sleep is disturbed due to developmental changes in the circadian rhythm and the natural process of reducing sleep drive during puberty [7,8]. On the other hand, environmental factors, such as excessive homework, increased number of extracurricular activities, intelligent device usage before bedtime, leaving for school early in the morning, and increased caffeine intake, shorten sleep time.

In the last few decades, interest in food’s impact on sleep has grown dramatically. The increased intake of fruits, vegetables, whole grains, milk, and dairy products is a significant factor in promoting sleep quality [9]. On the other side, caffeine (naturally found in some fruit, beans of coffee, cacao, and guarana plants) negatively correlates with sleep. Historically, the traditional method of caffeine intake was primarily via drinking coffee and tea, practiced mainly by adults. Children and adolescents, in these circumstances, were at lower risk of excessive caffeine ingestion because the specific taste limited their coffee preference. In the meantime, energy drinks rich in caffeine and sugar have become very popular, and because of their pleasing taste and wide availability, they have evolved into a vast threat to teenagers’ ingestion of high doses of caffeine.

Energy drinks (EDs) contain well-known substances (caffeine, taurine, and guarana) whose concentrations vary in a wide range [10], and alongside them, there are many other lesser-known ingredients whose effects on health carry a lot of controversy [11].

Caffeine is the primary psychoactive substance in most energy drinks. Depending on the manufacturer, the content of ingredients in EDs varies widely. The caffeine content of EDs varies from 50 to 505 mg per can [12]. A safe intake level for children and adolescents has not been officially determined; however, based on current scientific knowledge, they should not consume more than 100 mg per day [11]. Bearing in mind that most EDs contain about 160 mg of caffeine per can and that there is no age restriction on its purchase, there is a high risk of overconsumption and side effects appearing in the pediatric population [13]. Relatively mild adverse reactions (insomnia, nervousness, headaches, mood swings, upset stomach) are already manifested with the intake of about 200 mg of caffeine in comparison to the intake of more than 400 mg of caffeine, which increases the risk of severe adverse effects [14].

In addition to caffeine, EDs contain other substances: taurine, guarana, ginseng, L-carnitine, Ginkgo Biloba, B vitamins, milk thistle, and others [15]. The health impact of these components of ED has not been thoroughly investigated [14]. Research under controlled conditions determined that 200 mg of caffeine induces atrial flutter and atrial fibrillation [16]. Peake et al. [17] observed the development of rapid atrial fibrillation and cardiomyopathy after ingesting 575 mg of caffeine per day by consuming energy drinks. The study by Worthley et al., where young adults consumed EDs with 80 mg caffeine, 1000 mg taurine, and 600 mg glucuronolactone, found a significant increase in platelet aggregation and a decrease in endothelial function [18]. Caffeine intake in adolescents causes an increase in systolic and diastolic blood pressure [19]. At the same time, niacin intake in doses greater than 1 mg is seen as an inducer of acute hepatitis and the leading cause of the hepatotoxic potential of EDs [20]. The intake of 50 mg/day of pyridoxine, which is also a frequent constituent of EDs, is blamed for the appearance of peripheral neuropathy [21]. Psychiatric adverse effects include: symptoms of severe anxiety (6–8 cans EDs per day) [22]; worsening psychosis [23]; increased thought disorders in schizophrenic patients (intake of EDs at an amount of 10 mg/kg of caffeine or more) [24]; poor sleep, frequent headaches, and depressive symptoms [25]; and caffeine dependence [26].

As their reasons for consuming energy drinks, adolescents report a tendency to reduce fatigue and to improve the quality of sports and daily activities [27]. Interestingly, as the main reason for not consuming EDs, adolescents referred to parental or teacher prohibition, which means that they do not recognize EDs as unsafe by themselves [28].

The Academy of Pediatrics Committee on Nutrition in America and the Council on Sports Medicine and Fitness agreed that EDs could harm children’s health [29].

However, despite the above, the global ED market is rapidly growing. EDs are easily accessible and available with no age restriction [30]. Data regarding safe drinking quantities and caffeine concentration are hardly noticeable on product labels, yet well-designed packaging with some emotional branding attracts teenagers to buy the products [13]. It is not rare for athletes, despite being healthy lifestyle ambassadors, to promote and advertise EDs [31]. Studies have shown that adolescents who consume beverages rich in caffeine more frequently and in larger quantities fail to fulfill recommended length and sleep quality [32]. Scientists consider that caffeine from energy drinks can block adenosine and disrupt standard sleeping patterns [32]. The paradox is that adolescents find themselves in a vicious cycle and consequently expose themselves to the overconsumption of EDs to overcome feelings of tiredness caused by disrupted sleep.

The main aim of this research was to assess the relationship between regular energy drink intake (weekly or more frequently) and sufficient sleep (8 h or more) in male and female adolescents separately. We hypothesize that regular ED consumption is an independent risk factor negatively related to SS in both genders. Additional aims were to investigate the bond between health-related behaviors and sufficient sleep, stratified by gender.

## 2. Materials and Methods

### 2.1. Study Design and Participants

**Figure 1:**
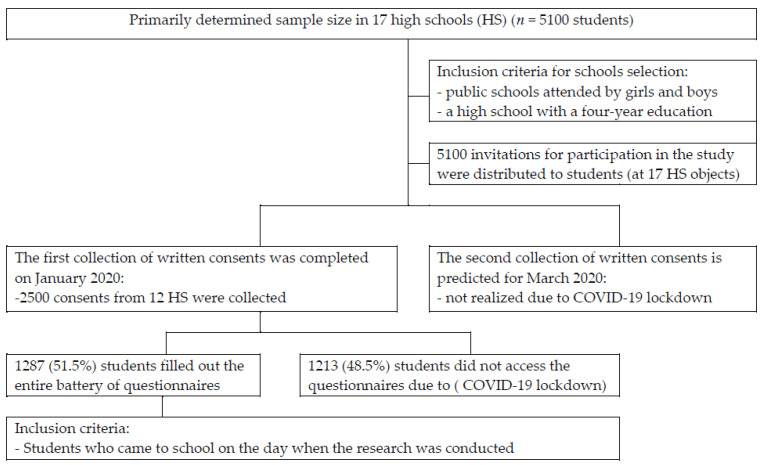
The process of the study group selection.

In this research, we used part of the data obtained from a cross-sectional study on the population of high school adolescents in Belgrade (Serbia) in the 2019/2020 school year. A representative sample determined the selection of 12 Belgrade high schools based on an insight acquired from the database of the Republic Institute for Statistics on secondary education of the Republic of Serbia [33]. For the sample’s representativeness, schools attended predominantly by girls or boys and high schools with a three-year duration were excluded from the selection. The process of the study group selection is shown in the graph in Figure 1.

Before the start of the research, each student from the selected schools and classes was given a copy of the respondent notification document with a detailed explanation of the study (Supplementary Materials File S1). Moreover, they were informed verbally by members of the research team about the following: the purpose of the study; the fact that the study was strictly confidential and that the complete anonymity of the participants was guaranteed; that participation in the study was based solely on goodwill, with no obligation to participate in the study till the end, and that if a student decided to withdraw from further participation for any reason, it would not be necessary to justify the withdrawal, nor would he bear any consequences as a result; and that all potential participants could access the information brochure, regardless of the decision to participate or not.

In this study, 1287 students aged 15 to 19 participated (37.4% male). Members of the research team distributed questionnaires during one school class. Participants filled out anonymous questionnaires individually.

### 2.2. Ethical Considerations

For the conduct of this study, we obtained the approval of the Ethics Committee of the Faculty of Medicine, University of Belgrade, number 1550/XI-38; the permission of the Ministry of Science and Education; the approval of school principals; and the written consent of parents/guardians and students who participated in the study.

### 2.3. Assessments

The respondents filled out a collection of questionnaires as part of the author’s doctoral dissertation surveys [34]. For the aim of this study, we extracted a specific set of questions to accomplish data for our research question. For all questionnaires used in the research, validity and reliability were verified.

#### 2.3.1. Sociodemographic Characteristics of the Sample

The first part of the data set was formed on the data obtained in response to the questions related to the sociodemographic characteristics of the sample (Supplementary Materials File S2). The study controlled for gender, parental education, and socioeconomic status. We collected data on gender and separately examined parental education with questions about the mother’s and father’s education levels. The offered answers were “High school” and “College.” We discussed their socioeconomic status through the question of whether the respondents’ parents could fulfill their children’s material needs. Answer options were “No” and “Yes.”

#### 2.3.2. Dietary Assessment

The second part of the data set was formed on the data obtained in response to the questions related to dietary assessment (Supplementary Materials File S2). We examined nutritional factors using a food frequency inventory that we designed following the basic framework from the 2012 Youth Adolescent Food Frequency Questionnaire [35] adapted for Serbian adolescents. Obtained data were analyzed through questions divided into two sets. The first set of questions related to a healthier diet (intake of fruits; vegetables; milk; fish; eggs; whole grain bread; and water), and the second set of questions was related to unhealthy eating habits: ingesting sodas, energy drinks, fast food, snacks, and adding extra salt to meals. We obtained data through the following questions: “How often do you eat fresh fruit?” (answers: 1—weekly or less; 2—every day); “How often do you eat fresh vegetables?” (answers: 1—weekly or less; 2—every day), “Do you drink milk?” (answers: 0—no; 1—yes); “How often do you eat fish?” (answers: 1—monthly or less; 2—weekly or more); “How often do you eat eggs? (answers: 1—monthly or less; 2—weekly or more); “Do you eat whole grain bread?” (answers: 0—no; 1—yes); “How much water do you drink per day?” (1—less than 2 L; 2—2 L or more); How many cups of coffee do you drink per day?” (answers: 1—two or less; 2—three or more); “Do you drink sodas?” (answers: 1—monthly or less; 2—weekly or more); “How often do you eat fast food?” (answers: 1—monthly or less; 2—weekly or more); “How often do you eat snacks?” (answers: 1—monthly or less; 2—weekly or more); “Do you add extra salt to your food?” (answers: 0—no; 1—yes).

#### 2.3.3. Energy Drink (ED)

Regular energy drink intake: In this study, the primary explanatory variable was the question of energy drink intake (weekly or more frequent). We formulated this variable as: “Do you drink energy drinks (1 piece = bottle/can 0.33 L)? The answers offered were: 0—never; 1—weekly; 2—every day; 3—a few times daily”. We recoded answers into a binary variable where 0 = no if respondents answered “never” and 1 = yes if responses were: weekly, every day, or a few times daily.

#### 2.3.4. Healthy Lifestyle Habits

The third part of the data set was formed on the data obtained in response to the questions related to healthy lifestyle habits. Based on the previous studies, we added two more sets of covariables to the third part of the questionnaire: the influence of a healthy lifestyle (physical activity and sufficient sleep) (Supplementary Materials File S2).

#### 2.3.5. Physical Activity

Physical activity was examined with the question: “How many times in the last seven days have you been physically active for at least one hour? (Sports, fitness/gym, long walk)?” (the offered answers were: 0—never; 1—once; 2—twice; 3—three times; 4—four times, 5—five times, 6—six times; 7—seven times). However, because of the appropriate criteria for a physically active lifestyle, according to the CDC [36], the answers “0, 1, 2, 3, 4, 5” were recoded into the binary variable “0—no” and answers “6 and 7” were recoded into “1—yes”.

#### 2.3.6. Sufficient Sleep (SS)

Sufficient sleep (SS) was the primary outcome variable in this study. The original question that defined this variable was: “How many hours do you usually sleep during the night” with five offered answers: 1—five hours and less; 2—six hours on average; 3—seven hours on average; 4—eight hours on average; and 5—nine hours or more”. Considering the recommendations [4] that adolescents aged 13 to 19 need a minimum of 8 h of sleep daily for proper psychophysical growth and development, the original answers were recoded into a binary variable. The numerical value 0 represents adolescents with insufficient sleep (shorter than 8 h) and 1 for adolescents with sufficient sleep (8 h or more).

#### 2.3.7. Unhealthy Lifestyle Habits and Mental Health Issues

The fourth part of the data sets was formed on the data obtained in response to the questions related to unhealthy lifestyle habits and mental health problems (Supplementary Materials File S2). The influence of unhealthy lifestyle habits (smoking, alcohol consumption, and drug use) was examined through the following questions separately: “Do you smoke”; “Do you consume alcohol?”; “Do you use drugs?” (offered answers for every question were: 0—no and 1—yes). Regarding self-reported mental health and sedative use, respondents were asked whether they were taking sedatives and whether they ever had symptoms of anxiety or depression (offered answers: 0—no; 1—yes).

### 2.4. Statistical Data Analysis

In descriptive statistics, we operated with percentage calculations. In inferential statistics, we used the chi-square test and binary logistic regression. The Pearson chi-square test examined the bivariate association between sociodemographic characteristics and SS, stratified by gender. We analyzed the relationship between SS and EDs as the primary explanatory variable and between SS and other characteristics of the sample withunivariate logistic regression. Finally, we used multiple logistic regression to investigate the association between SS and EDs in the presence of confounding factors previously detected as significant in univariate logistic regression. We constructed two models, one for male and one for female adolescents. The confidence interval was 95%, and data are presented as odds ratios and adjusted odds ratios. Variables were considered significant if the *p*-value was less than 0.05. All statistical analyses were performed using IBM SPSS Statistics for Windows, Version 22.0 (IBM Corp., Armonk, NY, USA).

## 3. Results

### 3.1. General Characteristics

In this study, 1287 adolescents aged 15 to 19 took participation. The gender distribution favored girls (62.6%) compared to boys (37.4%). We found that only 29.5% of respondents had a sufficient sleep (Table 1). SS was more frequent among boys (32.6%) than girls (27.6%), but this difference was not statistically significant (Table 1). According to the regular intake of energy drinks, 17,1% of adolescents consume EDs weekly or more, and boys do so more than girls (19.8% and 15.6%, respectively) (Table 1).

### 3.2. Dietary Factors

Considering the relationship between sufficient sleep and regular energy drink intake, we found a statistically significant negative correlation in both males (*p* < 0.001) and female (0.005) adolescents (Table 2).

In males, we found a statistically significant positive association between daily intake of fresh vegetables and water (2 L or more) and consuming milk. Regarding the group of females, there was an insignificant relationship between any foods and sufficient sleep (Table 2). Other factors of a healthy diet, such as weekly or more frequent consumption of fish and whole grain bread, were insignificantly positively connected with maintaining SS, in contrast to eggs, where increased intake negatively correlated with SS in males and females. Regarding unhealthy eating habits, we found a statistically insignificant positive association between SS with higher doses of daily coffee intake, more frequent snack consumption, and fast-food consumption in both genders. In contrast, we found an insignificant negative relationship between SS and the regular intake of sodas and the habit of adding extra salt to a meal (Table 2).

### 3.3. Physical Activity

We found that being physically active (PA) for at least one hour a day significantly raises the odds of SS in males. On the other hand, among females, a positive but insignificant correlation between PA and SS is revealed (Table 2).

### 3.4. Health-Risk Behaviors and Mental Issues

Regarding health-risk behaviors such as smoking cigarettes, drinking alcohol, and drug use, we found that those harmful health behaviors reduce the odds of SS in male adolescents, but only cigarette smoking showed statistical significance (Table 2). None of the unhealthy habits among girls did not show a significant relation to SS.

Regarding mental health problems, we found a strong negative correlation between self-reported sedative use and SS in both males and females (Table 2). On the other hand, the relation to self-reported anxious–depressive symptoms in males was not significant; this was the opposite to the female group of adolescents, where the connection between these mental issues and SS was statistically significant (Table 2).

Finally, through two multiple logistic regression models stratified by gender (Table 3), we examined the association between SS and EDs adjusted for confounding variables, which was previously recognized as significant in relation to SS in univariate logistic regression (Table 2).

In both models, male and female, we found a statistically significant connection between adjusted EDs and SS, confirming our hypothesis (Table 3). Regarding other potent confounding variables in the male group, the significant association found in univariant logistic regression (Table 2) remained stable concerning vegetables and water intake but vanished in terms of milk consumption, physical activity, smoking cigarettes, and sedative use. Significant confounding variables found in females, such as sedative use (Table 2), showed persistence, but on the other side, significance disappeared in the case of anxious–depressive symptoms (Table 3).

## 4. Discussion

The main aim of this study was to investigate the association between regular energy drink intake and sufficient sleep in a population-based sample of adolescents. Our study is the first in Serbia and the surrounding countries to examine this relationship.

Regarding our results, less than one-third (29.5%) of adolescents meet sufficient sleep requirements (Table 1). These numbers are higher than in the study by Hissing and colleagues in Norway, where only 15.2% of high school students sleep for 8 h or more [37]. Conversely, the percentages of adolescents who meet recommended sleep hours are higher in certain countries such as in America, Asia, and Europe [38,39], but still, it remains a huge problem even in those countries. As the possible cause of sleep shortage, adolescents point out stress, environmental noise, obligations that limit the time estimated for sleep, overthinking and worries before bedtime, no bedtime routines, and co-sleeping [40,41,42].

We also found that boys sleep for slightly more extended times than girls, but this was statistically insignificant. Findings from previous studies are inconsistent regarding gender differences in sleep duration. Some studies have shown that boys sleep for longer [43,44], and others show that girls do [45], while there are also studies with results showing that sleep duration between male and female teenagers is equal [46].

We did not notice in our study that a higher level of mothers’ or fathers’ education or satisfaction with material status in adolescence significantly improves sleep duration. However, this is different from Bøes et al.’s study, which showed that children from better-educated and well-off families have better odds of attaining SS [47].

According to our findings, around 17% (Table 1) of adolescents in Serbia stated that they consume at least one energy drink weekly. This prevalence is consistent with assessments of studies in Canada [48] and Australia [49], but less than estimated in the Netherlands [50] and the UK [51].

The present study showed that boys more frequently consume energy drinks than girls, which is in line with previous studies [52,53]. As we know, no study investigates the specifics of gender patterns concerning the intake of energy drinks. These differences could contribute to the fact that boys are more impulsive and prone to risky behavior while, at the same time, girls think more about the possible consequences of taking a risk [54].

Maybe we could interpret these gender differences, also, in light of the same differences in adults. Namely, the finding that adult men are frequent consumers of EDs is consistent in numerous studies [55,56]. Wimer and Levant concluded that ED consumption is associated with the ideology of the self-presentation of a robust masculine identity prone to risky behavior [57]. In addition, marketing strategies for energy drinks are closely related to extreme sports, where hidden messages allude to masculinity and apprize risky behavior [58].

Our findings showed a negative association between regular EDs and SS in both male and female adolescents. The connection between sleep and EDs is consistent and has been represented through numerous studies [32,59]. EDs contain caffeine, guarana, taurine, ginseng, sugar, and B vitamins, but among these are additional lesser-known ingredients [60]. Caffeine is the main ingredient of EDs [8], and scientists believe that the primary mechanism of ED’s effect on sleep occurs through it. Caffeine is an adenosine receptor antagonist. It supports the release of excitatory neurotransmitters and leads to increased alertness and reduced fatigue [61,62]. Additionally, other ED ingredients such as sugar, B vitamins, amino acids, and especially taurine may act synergistically and potentiate the effects of caffeine [63].

Furthermore, we examined dietary factors as possible confounders in the current study. Interestingly, we found sex differences regarding nutritional elements and SS. In the boys’ group, daily intake of fresh vegetables, 2 L water per day or more, and milk consumption significantly raised the odds for SS. However, after adjusting for other statistically significant covariates, fresh vegetables and daily water intake attenuated but stayed significant. On the same occasions, milk consumption lost its significance. On the other hand, in the group of females, there was no significant relationship between food and sufficient sleep. As we know, no studies in the literature explain these differences.

Interest in the role of dietary factors in sleep regulation has grown significantly in recent years [64]. Primarily, scientists explain the acting mechanisms of nutritional factors on sleep with the role of caffeine and melatonin. Caffeine is linked to shortening sleep and impaired sleep quality. On the other hand, melatonin, known as the sleep hormone, activates MT1 and MT2 receptors related to G-protein and induces sleep by reacting to a decrease in light intensity [65]. Melatonin, which facilitates falling asleep and improves sleep quality, is synthesized by the pineal gland but it is also found exogenously in food. It is known that exogenous melatonin from food also affects sleep.

Next, scientists hypothesized that the mechanism by which nutrition influences sleep is in the potential of certain nutrients to change the commensal microbiota by distorting the brain–gut–microbiota axis [66].

In light of that evidence, Imaki et al. [67] found that a higher intake of fruits or vegetables positively correlates with the length of sleep, while Zuraikat et al. [68] found a positive correlation with better sleep quality. The explanation is that fruits, vegetables, and cereal fibers are rich sources of gamma-aminobutyric acid (GABA). GABA inhibits nerve activity in the central nervous system, decreasing the latency to fall asleep and prolonging sleep.

Furthermore, leafy vegetables are rich in micronutrients that can affect sleep, such as tryptophan, potassium, magnesium, fiber, iron, calcium, vitamin C, lutein, zeaxanthin, choline, complex carbohydrates, and beta carotene [69]. Nisar et al. [70] also observed a connection between dairy products and sleep quality. Dairy products are a rich source of calcium and tryptophan, where calcium helps the brain use tryptophan to produce melatonin [71]. Dairy products are also a source of selenium, with antioxidant properties associated with better sleep [72]. Butanoic acid, found in cow’s milk, is associated with a reduced likelihood of difficulty maintaining sleep [73].

Other factors of a healthy diet in our study, such as the frequent consumption of fruit, fish, and whole grain bread, were in insignificant positive correlation with maintaining SS in males, in contrast to egg intake, of which increased intake had a negative relationship with SS. Regarding unhealthy eating habits, we found an insignificant positive relationship between male adolescents who intake higher doses of coffee and frequently eat snacks or fast food, and SS. On the other side, we found an insignificant negative relationship between boys who regularly consume sodas and have the routine of adding extra salt to a meal and SS (Table 2).

In light of physical activity in our study, we found that physically active boys have significantly higher odds of realizing SS. However, this connection disappears after adjustment for other potential confounding factors (energy drinks, vegetables, milk, water intake, smoking cigarettes, sedative use, and self-reported presence of anxious–depressive symptoms). Furthermore, physically active girls in our study also showed a positive but insignificant correlation with SS. Several studies have shown that adolescents with lower physical activity levels have shorter and poorer sleep quality [74,75]. A recent meta-analysis concluded that physically active persons are more likely to have adequate sleep duration, continuity of night sleep, and less frequent daytime sleepiness [76].

Furthermore, regarding health-risk behaviors (smoking cigarettes, drinking alcohol, and drug use), we found that these factors reduce the odds of SS in boys, but only smoking was statistically significant. Regarding the group of females, none of the health risk factors we examined were not in significant correlation with SS. The negative influence of alcohol on sleep has been well documented in numerous studies [77,78]. It is confirmed that alcohol causes depression in the central nervous system, slows down brain activity, and causes drowsiness. However, alcohol leads to sleep fragmentation and preterm awakening after falling asleep and can even cause sleep apnea [79]. Studies show that different psychoactive substances and sleep problems function in a two-way connection: substance abuse leads to sleep disruption and vice versa [80].

Regarding mental health problems, we found a strong negative correlation between self-reported sedative use and SS in both males and females (Table 2). However, after adjusting for other potential confounding factors (energy drinks, vegetables, milk, water intake, physical activity, smoking cigarettes, self-reported presence of anxious–depressive symptoms), this correlation in male adolescents was lost, but in females stayed significant. Anxious–depressive symptoms and SS in males were not in a statistically significant relationship, neither in univariate nor multivariate logistic regression. For females, we noticed that significance in univariate logistic regression was lost after adjusting for other significant confounders. In the large meta-analysis, Lovato and Gradisar showed that sleep disturbances, such as short sleep duration, awakening during sleep, prolonged sleep latency, and poor sleep efficiency are commonly reported as predictive factors of depressive or anxiety symptoms among adolescents [79]. Insomnia, insufficient sleep, or poor sleep quality may influence the development of mental disorders through genetic pathways involving polymorphisms in circadian clock genes and serotonin systems [80]. Insufficient sleep leads to the dysregulation of dopamine in the limbic and striatal areas, which causes malfunctioning of the reward system and increased susceptibility to mental disorders [80].

In summary, our study contributes to relatively insufficient literature on the association between ED and sleep in adolescence, when an adequate amount of sleep is essential to normal growth and development. Although there is clear evidence regarding the potentially harmful effects of EDs, the popularity of these drinks is growing unstoppably. This growth trend can be controlled only by the strength of scientific evidence, which urges authorities to put the importance of public health before corporate profits.

Our research can help educate and raise awareness about the dangers of ED consumption that young people are unfamiliar with. It is equally important to raise the awareness of parents and teachers about the hidden risks of colorful and attractive ED packaging placed nonchalantly next to other drinks on the market. The current study’s results may also contribute to school campaigns against unhealthy lifestyle habits of adolescents, which will, besides smoking, drugs, or alcohol, highlight the harmful effects of EDs.

## 5. Strengths and Limitations

The strength of this study lies in the fact that we examined the association between energy drink intake and sleep duration stratified by gender. Additionally, the relationship between SS and EDs was reviewed and confirmed by models that considered the potential confounders’ influence. Nonetheless, this study did have specific weaknesses: objective measures of sleep patterns in non-laboratory settings (actigraphy, bed sensors, non-invasive arm sensors) were not used. Additionally, serum caffeine concentration and the concentrations of different psychoactive substances were not explicitly determined which, if measured, could reduce self-report bias. Objective environmental factors that could affect sleep, for example, extracurricular activities in the late evening hours, early morning school duties, and high ecological noise were also not considered.

## 6. Conclusions

The results of this study verified our hypothesis that energy drink intake, as an independent risk factor, is negatively correlated with meeting recommended hours of sleep in adolescents. This correlation was found with boys as well as with girls, which is in line with previous studies. We also determined that boys who eat vegetables more regularly and drink larger amounts of water are more likely to accomplish recommended sleep length. On the contrary, girls who use sedatives are less likely to meet those requirements.

It is necessary to conduct further research to test the causal association between ED intake and SS, along with the characteristics of confounding variables concerning gender-related differences found in our study. The education of children, parents, and teachers about the potential health risks of ED consumption, on the one hand, and the adoption of effective laws and regulations that would limit ED distribution among the underaged, on the other hand, would bring a great deal of impact.

## Figures and Tables

**Table 1:** Bivariate association between: characteristics of the sample and gender (a); characteristics of the sample and sufficient sleep (b) stratified by gender (*n* = 1287).

Characteristics of the Sample						Gender (a)						Sufficient Sleep (b)						
Variables			Total			Male			Female			*p* *	Male			Female		
			*n*	%	*	*n*	%	*	*n*	%	*		No %	Yes %	*p* *	No %	Yes %	*p* *
Sex	Male		483	37.4									67.4	32.6		72.4	27.6	0.058
	Female		804	62.6														
PE	Father	HS	625	49.0		243	50.7	4	382	48.0		0.343	69.8	30.2	0.270	70.2	29.8	0.188
		College	650	51.0		236	49.3		414	52.0			65.1	34.9		74.3	25.7	
	Mother	HS	561	44.1		204	42.7	5	357	45.0		0.416	70.9	29.1	0.116	70.9	29.1	0.399
		College	710	55.9		274	5.3		436	55.0			64.1	35.9		73.6	26.4	
PFI	Unfulfilled needs		92	7.20		36	7.5	1	56	7.0	3	0.748	66.7	33.3	0.933	78.6	21.4	0.291
	Fulfilled needs		1191	92.8		446	92.3		745	93.0			67.3	32.7		72.0	28.0	
ED	No		1050	82.9	20	377	80.2	13	673	84.4	7	0.054	63.7	36.3	0.000	70.5	29.5	0.004
	Yes		217	17.1		93	19.8		124	15.6			82.8	17.2		83.1	16.9	
SS	No		903	70.5	6	322	67.4	5	581	72.4	1							
	Yes		378	29.5		156	32.6		222	27.6								

PE—parental education; PFI—perception of family income; ED—energy drinks; SS—sufficient sleep; HS—high school; *—missing response.

**Table 2:** The univariate logistic regression stratified by gender: personal factors and sleep sufficiency among adolescents (*n* = 1287).

Sufficient Sleep							
Dietary factors	**Main Explanatory Variable:**	**Male**			**Female**		
		**OR**	**95% C.I.**	***p*** **Value**	**OR**	**95% C.I.**	***p*** **Value**
	Energy drinks (Monthly or less)						
	Weekly or more	**0.365**	**(0.205–0.651)**	**0.001**	**0.488**	**(0.297–0.803)**	**0.005**
	Potential confounding variables:						
	Fresh fruits (Weekly or less)						
	Every day	1.366	(0.911–2.049)	0.132	1.297	(0.938–1.794)	0.115
	Fresh vegetables (Weekly or less)						
	Every day	**1.975**	**(1.311–2.975)**	**0.001**	1.286	(0.934–1.770)	0.124
	Milk (No)						
	Yes	**1.912**	**(1.115–3.277)**	**0.018**	1.259	(0.886–1.789)	0.200
	Fish (Monthly or less)						
	Weekly or more	1.213	(0.819–1.794)	0.335	1.043	(0.756–1.439)	0.797
	Eggs (Monthly or less)						
	Weekly or more	0.729	(0.441–1.206)	0.218	0.999	(0.694–1.436)	0.994
	Whole grains (No)						
	Yes	1.429	(0.945–2.161)	0.091	0.985	(0.714–1.359)	0.926
	Daily water intake (<2 L)						
	≥ than 2 L	**1.830**	**(1.225–2.734)**	**0.003**	1.179	(0.787–1.768)	0.425
	Sodas (Monthly or less)						
	Weekly or more	0.868	(0.586–1.287)	0.481	0.772	(0.566–1.054)	0.104
	Daily coffee intake (Two or less)						
	Three or more	1.285	(0.521–3.167)	0.586	0.997	(0.435–2.285)	0.994
	Snacks (Monthly or less)						
	Weekly	1.140	(0.752–1.730)	0.537	1.060	(0.752–1.493)	0.739
	Fast food (Monthly or less)						
	Weekly	0.875	(0.592–1.292)	0.501	1.294	(0.941–1.780)	0.113
	Extra salting food (No)						
	Yes	0.738	(0.474–1.151)	0.180	1.036	(0.726–1.478)	0.847
Physical activity	Physically active (No)						
	Yes	**1.621**	**(1.051–2.499)**	**0.029**	1.283	(0.760–2.166)	0.350
Harmful health behavioral habits and self-reported mental issues	Smoking (No)						
	Yes	**0.564**	**(0.328–0.969)**	**0.038**	1.004	0.671–1.502	0.985
	Alcohol use (No)						
	Yes	0.733	(0.494–1.089)	0.180	0.978	(0.712–1.343)	0.889
	Drugs use (No)						
	Yes	1.033	(0.347–3.076)	0.953	0.578	(0.124–2.695)	0.485
	Sedatives use (No)						
	Yes	**0.115**	**(0.015–0.869)**	**0.036**	**0.264**	**0.104–0.673**	**0.005**
	Anxious-depressive sympt. (No)						
	Yes	0.547	(0.285–0.776)	0.070	**0.572**	**(0.375–0.874)**	**0.010**

C.I.—confidence interval; OR—odds ratio; sympt.—symptoms; significant values are highlighted in bold.

**Table 3:** Multivariable logistic regression models by genders (*n* = 1287).

	Sufficient Sleep					
	Model I			Model II		
	Male			Female		
Variables	AOR	(95% C.I.)	*p*	AOR	(95% C.I.)	*p*
Energy drinks (No) Yes	**0.448**	**(0.243–0.824)**	**0.010**	**0.556**	**(0.331–0.934)**	**0.026**
Vegetables (Weekly or less) Every day	**1.883**	**(1.177–2.856)**	**0.007**			
Milk (Do not drink); Drink	1.632	(0.910–2.927)	0.100			
Water intake (<2 L/day); 2 L/day or more	**1.716**	**(1.095–2.690)**	**0.019**			
Physically active (No); Yes	1.137	(0.694–1,863)	0.610			
Smoking cigarettes (No) Yes	0.854	(0.471–1.549))	0.603			
Sedative use (No) Yes	0.164	(0.021–1.305)	0.088	**0.376**	**(0.143–0.989)**	**0.048**
Anxious- depressive symptoms (No) Yes				0.687	0.443–1.065	0.093

AOR— adjusted odds ratio; C.I.— confidence interval; significant values are highlighted in bold.

## Data Availability

Additional data are available from the corresponding author upon reasonable request.

## Supplementary Materials

The following supporting information can be downloaded at: https://www.mdpi.com/article/10.3390/nu14183813/s1, File S1: Explanation of the study; File S2: Questionnaires.

## References

[1] Cirelli C., Tononi G. (2008). Is sleep essential?. PLoS Biol..

[2] Silva E.M.B., Simões P.A.D., Macedo M.C.S.A., Duarte C.J., Silva M.D. (2018). Parents’ perception of the sleep habits and quality of preschool-aged children. Rev. Enferm. Ref..

[3] Brand S., Kirov R. (2011). Sleep and its importance in adolescence and in common adolescent somatic and psychiatric conditions. Int. J. Gen. Med..

[4] Paruthi S., Brooks L.J., D’Ambrosio C., Hall W., Kotagal S., Lloyd R.M., Malow B.A., Maski K., Nichols C., Quan S.F. (2016). Consensus statement of the American Academy of Sleep Medicine on the recommended amount of sleep for healthy children: Methodology and discussion. J. Clin. Sleep Med..

[5] Cappuccio F.P., Taggart F.M., Kandala N.-B., Currie A., Peile E., Stranges S., Miller M.A. (2008). Meta-analysis of short sleep duration and obesity in children and adults. Sleep.

[6] Yang C.K., Kim J.K., Patel S.R., Lee J.H. (2005). Age-related changes in sleep/wake patterns among Korean teenagers. Pediatrics.

[7] Crowley S.J., Acebo C., Carskadon M.A. (2007). Sleep, circadian rhythms, and delayed phase in adolescence. Sleep Med..

[8] Owens J.A., Weiss M.R. (2017). Insufficient sleep in adolescents: Causes and consequences. Minerva Pediatr..

[9] Córdova F.V., Barja S., Brockmann P.E. (2018). Consequences of short sleep duration on the dietary intake in children: A systematic review and metanalysis. Sleep Med. Rev..

[10] Clauson K.A., Shields K.M., McQueen C.E., Persad N. (2008). Safety issues associated with commercially available energy drinks. J. Am. Pharm. Assoc..

[11] Seifert S.M., Schaechter J.L., Hershorin E.R., Lipshultz S.E. (2011). Health effects of energy drinks on children, adolescents, and young adults. Pediatrics.

[12] Reissig C.J., Strain E.C., Griffiths R.R. (2009). Caffeinated energy drinks: A growing problem. Drug Alcohol Depend..

[13] Harris J.L., Munsell C.R. (2015). Energy drinks and adolescents: What’s the harm?. Nutr. Rev..

[14] Wolk B.J., Ganetsky M., Babu K.M. (2012). Toxicity of energy drinks. Curr. Opin. Pediatr..

[15] Babu K.M., James C.R., Lewander W. (2008). Energy drinks: The new eye-opener for adolescents. Clin. Ped. Emerg. Med..

[16] Dobmeyer D.J., Stine R.A., Leier C.V., Greenberg R., Schaal S.F. (1983). The arrhythmogenic effects of caffeine in human beings. N. Engl. J. Med..

[17] Peake S.T., Mehta P.A., Dubrey S.W. (2007). Atrial fibrillation-related cardiomyopathy: A case report. J. Med. Case Rep..

[18] Worthley M.I., Prabhu A., De Sciscio P., Schultz C., Sanders P., Willoughby S.R. (2010). Detrimental effects of energy drink consumption on platelet and endothelial function. Am. J. Med..

[19] Savoca M.R., Evans C.D., Wilson M.E., Harshfield G.A., Ludwig D.A. (2004). The association of caffeinated beverages with blood pressure in adolescents. Arch. Pediatr. Adolesc. Med..

[20] Vivekanandarajah A., Ni S., Waked A. (2011). Acute hepatitis in a woman following excessive ingestion of an energy drink: A case report. J. Med. Case Rep..

[21] Schaumburg H., Kaplan J., Windebank A., Vick A., Rasmus S., Pleasure D., Brown M.J. (1983). Sensory neuropathy from pyridoxine abuse. A new megavitamin syndrome. N. Engl. J. Med..

[22] Berigan T. (2005). An anxiety disorder secondary to energy drinks: A case report. Psychiatry (Edgmont).

[23] Menkes D.B. (2011). Transient psychotic relapse temporally related to ingestion of an energy drink. Med. J. Aust..

[24] Lucas P.B., Pickar D., Kelsoe J., Rapaport M., Pato C., Hommer D. (1990). Effects of the acute administration of caffeine in patients with schizophrenia. Biol. Psychiatry.

[25] Smaldone A., Honig J.C., Byrne M.W. (2007). Sleepless in America: Inadequate sleep and relationships to health and well being of our nation’s children. Pediatrics.

[26] Bernstein G.A., Carroll M.E., Thuras P.D., Cosgrove K.P., Roth M.E. (2002). Caffeine dependence in teenagers. Drug Alcohol Depend..

[27] Costa B.M., Hayley A., Miller P. (2014). Young adolescents’ perceptions, patterns, and contexts of energy drink use. A focus group study. Appetite.

[28] Holubcikova J., Kolarcik P., Geckova A.M., Van Dijk J., Reijneveld S. (2016). Lack of parental rules increases the risk for high intake of soft and energy drinks in adolescents. Eur. J. Public Health.

[29] Committee on Nutrition and the Council on Sports Medicine and Fitness (2011). Clinical report—sports drinks and energy drinks for children and adolescents: Are they appropriate?. Pediatrics.

[30] Hodge J.G., Scanlon M., Corbe A., Sorensen A. (2011). The consumable vice: Caffeine, public health, and the law. J. Contemp. Health L.

[31] Emond J.A., Sargent J.D., Gilbert-Diamond D. (2015). Patterns of energy drink advertising over US television networks. J. Nutr. Educ. Behav..

[32] Sampasa-Kanyinga H., Hamilton H.A., Chaput J.P. (2018). Sleep duration and consumption of sugar-sweetened beverages and energy drinks among adolescents. Nutrition.

[33] Statistical Office of the Republic of Serbia High/Secondary School Attendance. https://www.stat.gov.rs/en-us/oblasti/obrazovanje/srednje-obrazovanje/.

[34] Tomanić M.S. (2021). Prevalence of and Risk Factors for Tinnitus among Adolescents in an Urban Environment. Ph.D. Thesis.

[35] Harvard T.H. (2012). Chan School of Public Health Nutrition Department’s File Download Site: Youth Adolescent Food Frequency Questionnaire. https://regepi.bwh.harvard.edu/health/KIDS/files/02.%202012%20YOUTH%20ADOLESCENT%20FOOD%20FREQUENCY%20QUESTIONNAIRE.pdf.

[36] Center for Disease Control and Prevention (CDC) Physical Activity Guidelines for School-Aged Children and Adolescents. https://www.cdc.gov/healthyschools/physicalactivity/guidelines.htm#:~:text=Children%20and%20adolescents%20ages%206%20through%2017%20years%20should%20do,to%2Dvigorous%20physical%20activity%20daily.

[37] Hysing M., Pallesen S., Stormark K.M., Lundervold A.J., Sivertsen B. (2013). Sleep patterns and insomnia among adolescents: A population-based study. J. Sleep Res..

[38] Gariepy G., Danna S., Gobina I., Rasmussen M., Gaspar de Matos M., Tynjala J., Janssen I., Kalman M., Villeruša A., Husarova D. (2020). How are adolescents sleeping? Adolescent sleep patterns and sociodemographic differences in 24 European and North American Countries. J. Adolesc. Health.

[39] Gradisar M., Gardner G., Dohnt H. (2011). Recent worldwide sleep patterns and problems during adolescence: A review and meta-analysis of age, region, and sleep. Sleep Med..

[40] Brown F.C., Buboltz W.C., Soper B. (2002). Relationship of sleep hygiene awareness, sleep hygiene practices, and sleep quality in university students. Behav. Med..

[41] Forquer L.M., Camden A.E., Gabriau K.M., Johnson C.M. (2008). Sleep patterns of college students at a public university. J. Am. Coll. Health.

[42] Lund H.G., Reider B.D., Whiting A.B., Prichard J.R. (2010). Sleep patterns and predictors of disturbed sleep in a large population of college students. J. Adolesc. Health.

[43] Sarchiapone M., Mandelli L., Carli V., Iosue M., Wasserman C., Hadlaczky G., Hoven C.W., Apter A., Balazs J., Bobes J. (2014). Hours of sleep in adolescents and its association with anxiety, emotional concerns, and suicidal ideation. Sleep Med..

[44] Olds T., Blunden S., Petkov J., Forchino F. (2010). The relationships between sex, age, geography and time in bed in adolescents: A meta-analysis of data from 23 countries. Sleep Med. Rev..

[45] Keyes K.M., Maslowsky J., Hamilton A., Schulenberg J. (2015). The great sleep recession: Changes in sleep duration among US adolescents, 1991–2012. Pediatrics.

[46] Dalmases M., Benítez I.D., Mas A., Garcia-Codina O., Medina-Bustos A., Escarrabill J., Saltó E., Buysse D.J., Roure N., Sánchez-de-la-Torre M. (2018). Assessing sleep health in a European population: Results of the catalan health survey 2015. PLoS ONE.

[47] Bøe T., Hysing M., Stormark K.M., Lundervold A.J., Sivertsen B. (2012). Sleep problems as a mediator of the association between parental education levels, perceived family economy and poor mental health in children. J. Psychosom. Res..

[48] Reid J.L., McCrory C., White C.M., Martineau C., Vanderkooy P., Fenton N., Hammond D. (2017). Consumption of caffeinated energy drinks among youth and young adults in Canada. Prev. Med. Rep..

[49] Costa B.M., Hayley A., Miller P. (2016). Adolescent energy drink consumption: An Australian perspective. Appetite.

[50] Gambon D.L., Brand H.S., Boutkabout C., Levie D., Veerman E.C. (2011). Patterns in consumption of potentially erosive beverages among adolescent school children in the Netherlands. Int. Dent. J..

[51] Brunton G., Kneale D., Sowden A., Sutcliffe K., Thomas J. (2019). Caffeinated Energy Drinks and Effects in UK Young People: A Secondary Analysis of Population-Level Datasets.

[52] Mansour B., Amarah W., Nasralla E., Elias N. (2019). Energy drinks in children and adolescents: Demographic data and immediate effects. Eur. J. Pediatr..

[53] Martins A., Ferreira C., Sousa D., Costa S. (2018). Consumption Patterns of Energy Drinks in Portuguese Adolescents from A City in Northern Portugal. Acta Med. Port..

[54] Reniers R.L., Murphy L., Lin A., Bartolome S.P., Wood S.J. (2016). Risk perception and risk-taking behaviour during adolescence: The influence of personality and gender. PLoS ONE.

[55] Berger L.K., Fendrich M., Chen H.-Y., Arria A.M., Cisler R.A. (2011). Sociodemographic correlates of energy drink consumption with and without alcohol: Results of a community survey. Addict. Behav..

[56] Park S., Onufrak S., Blanck H.M., Sherry B. (2013). Characteristics associated with consumption of sports and energy drinks among US adults: National Health Interview Survey, 2010. J. Acad. Nutr. Diet.

[57] Wimer D.J., Levant R.F. (2013). Energy drink use and its relationship to masculinity, jock identity, and fraternity membership among men. Am. J. Mens. Health.

[58] Inchley J., Currie D., Cosma A., Samdal O. (2018). Health Behaviour in School-Aged Children (HBSC) Study Protocol: Background, Methodology and Mandatory Items for the 2017/2018 Survey. http://www.hbsc.org/methods/.

[59] Park S., Lee Y., Lee J.H. (2016). Association between energy drink intake, sleep, stress, and suicidality in Korean adolescents: Energy drink use in isolation or in combination with junk food consumption. Nutr. J..

[60] Larson N., DeWolfe J., Story M., Neumark-Sztainer D. (2014). Adolescent consumption of sports and energy drinks: Linkages to higher physical activity, unhealthy beverage patterns, cigarette smoking, and screen media use. J. Nutr. Educ. Behav..

[61] Lohsoonthorn V., Khidir H., Casillas G., Lertmaharit S., Tadesse M.G., Pensuksan W.C., Rattananupong T., Gelaye B., Williams M.A. (2013). Sleep quality and sleep patterns in relation to consumption of energy drinks, caffeinated beverages, and other stimulants among Thai college students. Sleep Breath.

[62] Bjorness T.E., Greene R.W. (2009). Adenosine and sleep. Curr. Neuropharmacol..

[63] Lin F.J., Pierce M.M., Sehgal A., Wu T., Skipper D.C., Chabba R. (2010). Effect of taurine and caffeine on sleep-wake activity in Drosophila melanogaster. Nat. Sci. Sleep.

[64] Shilo L., Sabbah H., Hadari R., Kovatz S., Weinberg U., Dolev S., Dagan Y., Shenkman L. (2002). The effects of coffee consumption on sleep and melatonin secretion. Sleep Med..

[65] Peuhkuri K., Sihvola N., Korpela R. (2012). Dietary factors and fluctuating levels of melatonin. Food Nutr. Res..

[66] Yan R., Andrew L., Marlow E., Kunaratnam K., Devine A., Dunican I., Christophersen C. (2021). Dietary Fibre Intervention for Gut Microbiota, Sleep, and Mental Health in Adults with Irritable Bowel Syndrome: A Scoping Review. Nutrients.

[67] Imaki M., Hatanaka Y., Ogawa Y., Yoshida Y., Tanada S. (2002). An epidemiological study on relationship between the hours of sleep and life style factors in Japanese factory workers. J. Physiol. Anthropol. Appl. Human Sci..

[68] Zuraikat F.M., Makarem N., Liao M., St-Onge M.P., Aggarwal B. (2020). Measures of poor sleep quality are associated with higher energy intake and poor diet quality in a diverse sample of women from the go red for women strategically focused research network. J. Am. Heart Assoc..

[69] Grandner M.A., Jackson N., Gerstner J.R., Knutson K.L. (2014). Sleep symptoms associated with intake of specific dietary nutrients. J. Sleep Res..

[70] Nisar M., Mohammad R.M., Arshad A., Hashmi I., Yousuf S.M., Baig S. (2019). Influence of Dietary Intake on Sleeping Patterns of Medical Students. Cureus.

[71] Kennedy D.O. (2016). B Vitamins and the Brain: Mechanisms, Dose and Efficacy—A Review. Nutrients.

[72] Zeng Y., Yang J., Du J., Pu X., Yang X., Yang S., Yang T. (2014). Strategies of functional foods promote sleep in human being. Curr. Signal Transduct. Ther..

[73] Gillis B.T., El-Sheikh M. (2019). Sleep and adjustment in adolescence: Physical activity as a moderator of risk. Sleep Health.

[74] Kaldenbach S., Leonhardt M., Lien L., Bjærtnes A.A., Strand T.A., Holten-Andersen M.N. (2022). Sleep and energy drink consumption among Norwegian adolescents—A cross-sectional study. BMC Public Health.

[75] Kredlow M.A., Capozzoli M.C., Hearon B.A., Calkins A.W., Otto M.W. (2015). The efects of physical activity on sleep: A metaanalytic review. J. Behav. Med..

[76] Angarita G.A., Emadi N., Hodges S., Morgan P.T. (2016). Sleep abnormalities associated with alcohol, cannabis, cocaine, and opiate use: A comprehensive review. Addict. Sci. Clin. Pract..

[77] Kwon M., Park E., Dickerson S.S. (2019). Adolescent substance use and its association to sleep disturbances: A systematic review. Sleep Health.

[78] Pieters S., Burk W.J., Van der Vorst H., Dahl R.E., Wiers R.W., Engels R.C. (2015). Prospective relationships between sleep problems and substance use, internalizing and externalizing problems. J. Youth Adolesc..

[79] Lovato N., Gradisar M. (2014). A meta-analysis and model of the relationship between sleep and depression in adolescents: Recommendations for future research and clinical practice. Sleep Med. Rev..

[80] Blake M.J., Trinder J.A., Allen N.B. (2018). Mechanisms underlying the association between insomnia, anxiety, and depression in adolescence: Implications for behavioral sleep interventions. Clin. Psychol. Rev..

