# Supplementary material for: Energy Drinks and Sleep among Adolescents
Source: Nutrients. 2022 Sep 15;14(18):3813. doi: 10.3390/nu14183813 (PMC9502542; doi:10.3390/nu14183813)
Supplement: Supplementary file 1 [file nutrients-14-03813-s001.zip › nutrients-1884153-supplementary.pdf]

File S1. Explanation of the study.

МЕДИЦИНСКИ ФАКУЛТЕТ УНИВЕРЗИТЕТА У БЕОГРАДУ

Предмет: Истраживање преваленције и фактора ризика за тинитус код адолесцената у градској средини

Поштовани,

Позивамо Вас да узмете учешће у студији истраживача са Медицинског факултета Универзитета у Београду о преваленцији и факторима ризика за тинитус код адолесцената у градској средини. Својим учешћем у овом истраживању дaћете значајан допринос прикупљању података о учесталости јављања тинитуса (зујања у ушима) код младих и бољем разумевању фактора ризика који доприносе настанку овог здравственог проблема.

Истраживањем је планиран обухват од око 5100 ученика, по 300 из 17 одабраних репрезентативних средњих школа, што ће бити до сада највећа студија о тинитусу на популацији адолесцената спроведена у свету.

Прикупљање података ће се вршити путем анонимних упитника с циљем откривања фактора ризика за настанак тинитуса, присуство и одређивање озбиљности тинитуса, утицај на квалитет живота, ставове младих према буци, мерење буке у школској средини, мерење телесне тежине, висине, крвног притиска и испитивање слуха.

Сви резултати који буду добијени попуњавањем упитника и другим мерењима биће чувани уз најстроже придржавање одредби Закона о заштити података, а подаци ће бити коришћени тако да идентификација идентитета испитаника и повезивање са одговорима / мерењима ни у ком случају неће бити могућа.

Уколико имате било каквих питања и недоумица желите да добијете више информација везано за наше истраживање стојимо Вам на располагању. Можете нас контактирати следећим путем: на број телефона 0113612762 или на е-маил: *milena.tomanic@med.bg.ac.rs*.

Срдачно Вам се захваљујемо на времену које сте издвојили да прочитате ово обавештење. Својим учешћем у истраживању дајете драгоцени допринос у стицању нових научних сазнања везаних за проблем зујања у ушима код младих. Резултати ове студије имају за циљ јачање постојећих и подршку развоју нових превентивних програма деловања на факторе ризика из животне средине који утичу на све присутнију појаву овог здравственог проблема у млађој популацији.

Истраживање се спроводи у оквиру пројетка бр. 175078 финансираног од стране Министарства за просвету, науку и технолошки развој Републике Србије које је дало сагласност за спровођење овог истраживања (одлука у прилогу).

С поштовањем,
Асистент др Милена Томанић

File S2. Questionnaires.

I PART: THE SOCIODEMOGRAPHIC CHARACTERISTICS

1. Gender: 1—Male; 2—Female
2. Paternal education: 1—High school; 2—College
3. Maternal education: 1—High school; 2vCollege
4. Do you think that your parents can fulfill all your financial needs? 0—No; 1—Yes

II PART: DIETARY ASSESSMENT

1. How often do you eat fresh fruits? 1—Monthly or less; 2—Weekly or more
2. How often do you eat fresh vegetables? 1—Monthly or less; 2—Weekly or more
3. Do you drink milk? 0—No; 1—Yes
4. How often do you eat fish? 1—Monthly or less; 2—Weekly or more
5. How often do you eat eggs? 1—Monthly or less; 2—Weekly or more
6. Do you eat whole grain bread? 0—No; 1—Yes
7. How much water do you drink per day? 1—Less than 2l; 2—2l or more
8. Do you drink sodas? 1—Monthly or less; 2—Weekly or more
9. How often do you eat fast food? 1—Monthly or less; 2—Weekly or more
10. How often do you eat snacks? 1—Monthly or less; 2—Weekly or more
11. Do you add extra salt to your food? 0—No; 1—Yes
12. How many cups of coffee do you drink per day? 1—Two or less; 2—Three or more
13. Do you drink energy drinks? 0—No; 1—Weekly; 2—Every day; 3—Few times daily [1]

III PART: HEALTHY LIFESTYLE HABITS

1. How many times in the last seven days have you been physically active for at least one hour? (Sports, fitness/gym, long walk)? 0—Never; 1—Once; 2—Twice; 3—Three times; 4—Four times, 5—Five times, 6—Six times; 7—Seven times [2]
2. How many hours do you usually sleep during the night?" 1—Five hours and less; 2—Six hours on average; 3—Seven hours on average; 4—Eight hours on average; 5—Nine hours or more [3]

IV PART: UNHEALTHY LIFESTYLE HABITS AND MENTAL HEALTH PROBLEMS

1. Do you smoke? 0—No and 1—Yes
2. Do you consume alcohol? 0—No and 1—Yes
3. Do you use drugs? 0—No; 1—Yes
4. Do you take tranquilizers? 0. No 1. Yes
5. Have you ever had symptoms of anxiety or depression 0. No 1. Yes

Explanation:

[1] For the purpose of binary logistic regression, we had to recode the answers: 0—No if respondents answered Never" and 1—Yes if responses were: weekly, every day, or a few times daily.

[2] The answers "0, 1, 2, 3, 4, 5" were recoded into the binary variable "0—No" and answers "6 and 7" "1—Yes", because of the appropriate criteria for a physically active life-style according to CDC, the answers "0, 1, 2, 3, 4, 5" were recoded into the binary variable "0—No" and answers "6 and 7" and "1—Yes".

[3] Considering the recommendations, the original answers were recoded into a binary variable. The numerical value 0 represents adolescents with insufficient sleep (shorter than 8 hours) and 1 for adolescents with sufficient sleep (8 hours or more).
